# Sexual dimorphism of acute doxorubicin-induced nephrotoxicity in C57Bl/6 mice

**DOI:** 10.1371/journal.pone.0212486

**Published:** 2019-02-20

**Authors:** Marianne K. O. Grant, Davis M. Seelig, Leslie C. Sharkey, Wan S. V. Choi, Ibrahim Y. Abdelgawad, Beshay N. Zordoky

**Affiliations:** 1 Department of Experimental and Clinical Pharmacology, College of Pharmacy, University of Minnesota, Minneapolis, Minnesota, United States of America; 2 Department of Veterinary Clinical Sciences, College of Veterinary Medicine, University of Minnesota, St. Paul, Minnesota, United States of America; National Institutes of Health, UNITED STATES

## Abstract

Doxorubicin (DOX) is a chemotherapeutic agent that has been reported to cause nephrotoxicity in rodent models and to a lesser degree in cancer patients. Female rodents have been shown to be protected against several features of DOX-induced nephrotoxicity. Nevertheless, the underlying mechanisms of this sexual dimorphism are not fully elucidated. Therefore, in the current study, we investigated the sex and time-dependent changes in pathological lesions as well as apoptotic and fibrotic markers in response to acute DOX-induced nephrotoxicity. We also determined the effect of acute DOX treatment on the renal expression of the sexually dimorphic enzyme, soluble epoxide hydrolase (sEH), since inhibition of sEH has been shown to protect against DOX-induced nephrotoxicity. Acute DOX-induced nephrotoxicity was induced by a single intra-peritoneal injection of 20 mg/kg DOX to male and female adult C57Bl/6 mice. The kidneys were isolated 1, 3 and 6 days after DOX administration. Histopathology assessment, gene expression of the apoptotic marker, *BAX*, protein expression of the fibrotic marker, transforming growth factor-β (TGF-β), and gene and protein expression of sEH were assessed. DOX administration caused more severe pathological lesions as well as higher induction of the apoptotic and fibrotic markers in kidneys of male than in female mice. Intriguingly, DOX inhibited sEH protein expression in kidneys of male mice sacrificed at 3 and 6 days following administration, suggesting that induction of sEH is not necessary for acute DOX-induced nephrotoxicity. However, DOX-induced inhibition of renal sEH in male mice may protect the kidney from further DOX-induced injury in a negative feedback mechanism. We also observed lower constitutive expressions of TGF-β and sEH in the kidney of female mice which may contribute, at least in part, to sexual dimorphism of DOX-induced nephrotoxicity.

## Introduction

Doxorubicin (DOX) is an effective chemotherapeutic agent that is widely used to treat several hematological and solid tumors in pediatric and adult cancer patients. However, its use is limited by its marked cardiotoxicity. In addition to cardiotoxicity, DOX has been reported to cause nephrotoxicity in cancer patients [1, 2] and in experimental animals [3, 4]. Indeed, DOX has been widely used as a rodent model of proteinuric nephropathy leading to renal fibrosis [5]. We and others have demonstrated that DOX-induced nephrotoxicity is more severe in male than in female adult rodents [3, 6]. However, the mechanisms underlying this sexual dimorphism are not fully elucidated.

Soluble epoxide hydrolase (sEH) is an important enzyme responsible for converting the protective epoxyeicosatrienoic acid (EET) metabolites of arachidonic acid to the less biologically active metabolites, dihydroxyeicosatrienoic acids (DHETs) [7]. Importantly, inhibition of sEH protected against DOX-induced nephropathy in male mice and rats [8, 9], suggesting that sEH is detrimental in the pathogenesis of DOX-induced nephropathy. *Ephx2* gene, which encodes the sEH protein, is a sexually dimorphic gene regulated by sex hormones [10]. The constitutive expression and activity of sEH have been demonstrated to be higher in the kidney and liver of male rodents [11, 12]. Nevertheless, it is not known whether there is a sex difference in DOX-induced regulation of sEH, since the effect of DOX on sEH expression has never been reported in female experimental animals. Therefore, in the current study, we determined the effect of acute DOX administration on sEH expression in the kidney of male and female C57Bl/6N mice. Our findings reveal important sex- and time-dependent differences in constitutive and DOX-induced regulation of sEH in the kidney, which may explain the sexual dimorphism of DOX-induced nephrotoxicity.

## Materials and methods

### Animals

The Institutional Animal Care and Use Committee (IACUC) at the University of Minnesota has approved all procedures involving animals for this specific study. Male (n = 41) and female (n = 34) C57Bl/6 mice were purchased from Charles River Laboratories (Raleigh, NC) at twelve weeks of age and given an acclimation period of one week. Mice were then administered either 20 mg/kg DOX by intraperitoneal (IP) injection (DOX group) or equivalent volume of sterile normal saline (Control group) as we previously described [13]. Mice were humanely euthanized 1 day (8 male-control, 8 male-DOX, 8 female-control, and 8 female-DOX), 3 days (4 male-control, 5 male-DOX, 4 female-control, and 4 female-DOX), or 6 days (6 male-control, 4 male-DOX, 5 female-control, and 5 female-DOX) after DOX or saline administration. Mortality was observed in the male-DOX groups followed for 3 days (1 out of 6 male-DOX mice) and 6 days (5 out of 9 male-DOX mice) after DOX administration as we previously reported [13]. Additional experiments were performed using C57Bl/6 mice that were castrated (4 male), ovariectomized (4 female) or sham-operated (4 male, 4 female) at 4 weeks of age by Charles River Laboratories. Gonadectomized and sham-operated mice were humanely euthanized at 13 weeks of age. At the experimental end point, mice from all groups were euthanized by decapitation under isoflurane anesthesia. Thereafter, terminal blood was collected, and kidneys were harvested, washed in ice-cold phosphate buffered saline solution, flash frozen in liquid nitrogen, and stored at -80°C until further analysis.

### Serum creatinine

Terminal blood was collected and allowed to clot at room temperature for 20 minutes. Blood was centrifuged at 4000 rpm for 30 minutes at 4°C, serum was collected and stored at -80°C until use. Fifteen μl of serum was used to determine serum creatinine levels in duplicates following manufacturer’s instructions using the Cayman Chemical Creatinine (Serum) Colorimetric Assay kit (number 700460, Cayman Chemical, Ann Arbor, MI).

### Histopathology

Tissue sections were collected at the same level of the left kidney from mice euthanized 24 hours, 3 and 6 days following DOX or saline administration. Sections were fixed in 10% neutral buffered formalin, processed and embedded in paraffin using standard methods. Thereafter, four-micron tissue sections were cut and stained with hematoxylin and eosin (H&E) or trichrome stain. Histopathologic evaluation was performed by a board certified veterinary pathologist who was blinded to the experimental group. Each stained hematoxylin and eosin stained section was examined for (a) interstitial cellularity (e.g., inflammation and fibrosis), (b) necrosis, (c) glomerular pathology (e.g., increased cellularity, matrix deposition, and capsular thickening), and (d) tubular pathology (e.g., epithelial loss, degeneration, and hyperplasia). Interstitial cellularity, which comprised inflammation or fibrosis, was assessed as absent, minimal interstitial infiltration, mild interstitial infiltration, moderate interstitial infiltration, marked interstitial infiltration. Necrosis, if present, was assessed based upon the estimated percentage of affected tissue area. For glomerular and tubular pathology, sections were assessed based upon the severity of the change (minimal, mild, moderate, or severe) and an estimated percentage of affected glomeruli. Each lesion was scored separately on 0–4 scale and the total score of renal damage was calculated as the sum of individual scores.

### Real-time polymerase chain reaction (PCR)

Total RNA from the frozen tissues was isolated and quantified, first-strand cDNA was synthesized, and quantitative analysis of specific mRNA expression was performed by real time-PCR as we previously described [13]. The following primers were used for *Ephx2* (F: GAA AGG ATT CAC AAC ATG CAT TG and R: GGC CAG GCT GTC TCT CTT GTC) and *BAX* (F: AGC AAA CTG GTG CTC AAG GC and R: CCA CAA AGA TGG TCA CT GTC). We used *β-actin* as a house-keeping gene (F: TAT TGG CAA CGA GCG GTT CC and R: GGC ATA GAG GTC TTT ACG GAT GTC).

### Protein extraction and western blotting

Frozen kidney tissue was homogenized as described previously [14] and protein concentration was determined using the Pierce bicinchoninic acid (BCA) protein assay kit (number 23255; Pierce, Thermo Fisher Scientific, Rockford, IL). 50 μg of protein was separated by sodium dodecyl sulfate-polyacrylamide gel electrophoresis (SDS-PAGE) and electro-transferred onto nitrocellulose membranes as previously described [15]. Mouse monoclonal sEH antibody (catalog 166961; 1:1000 dilution) was purchased from Santa Cruz Biotechnology (Dallas, TX). Rabbit polyclonal antibodies against TGF-beta (catalog 3711; 1:500 dilution) and alpha-tubulin (catalog 2144; 1:1000 dilution) were purchased from Cell Signaling (Danvers, MA). Secondary anti-mouse or anti-rabbit conjugated to HRP were purchased from Cell Signaling (catalog 7076; 1:1000 dilution) and Jackson ImmunoResearch (catalog 111-035-144; 1:10,000 dilution; West Grove, PA), respectively. Bands were quantified using ImageJ software (National Institutes of Health, Bethesda, MD) and normalized to alpha-tubulin protein levels as a loading control.

### Statistical analysis

Data were analyzed using the GraphPad Prism software (version 7.04) for Windows, La Jolla California USA, www.graphpad.com. Data shown are mean ± SEM. Comparisons among different sex and treatment groups were performed by 2-way Analysis of Variance (ANOVA), followed by Tukey’s multiple comparison test as a post-hoc analysis. Unpaired student t-test was used to compare between two groups. Histopathologic grading of lesions is presented as individual scores and their median with the interquartile range. Statistical analysis for histopathologic grading was performed using Kruskal-Wallis non-parametric test. A probability (P) value of < 0.05 was taken to indicate statistical significance.

## Results

### Females are less sensitive to acute DOX-induced nephrotoxicity

**Fig 1:**
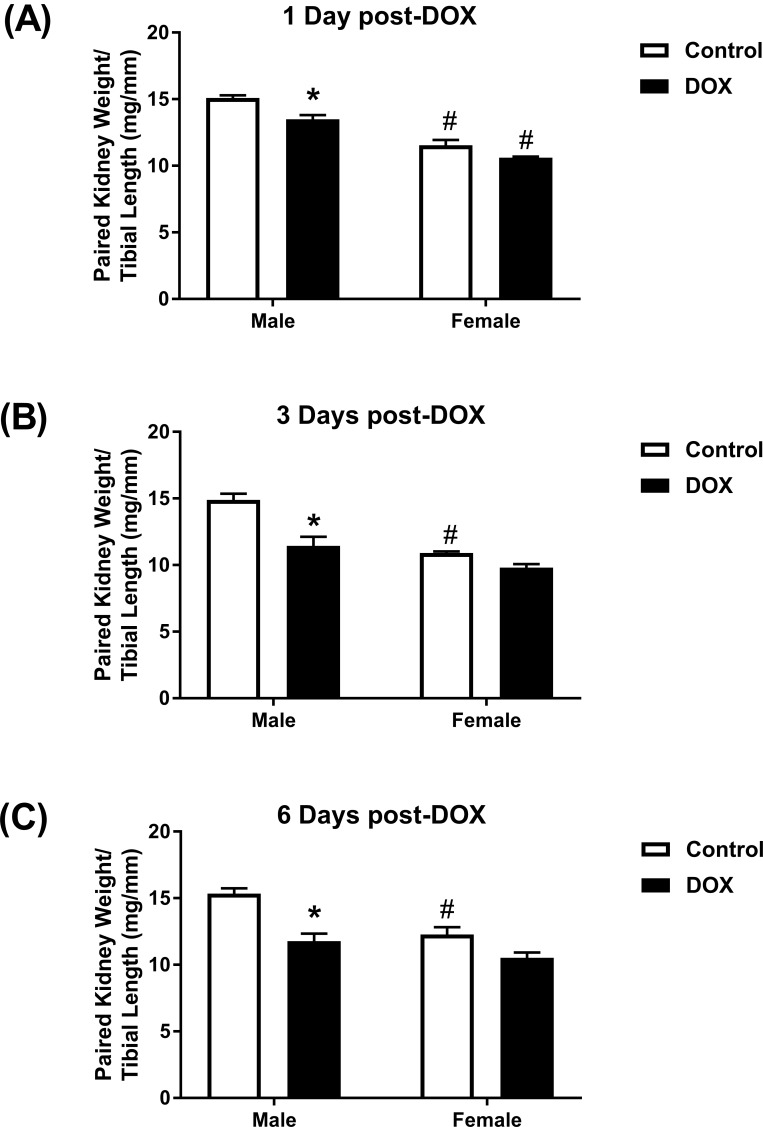
Effect of acute DOX administration on kidney weight. A single intraperitoneal injection of 20 mg/kg DOX or equivalent volume of sterile saline was administered to adult male and female C57Bl/6 mice. Paired kidney weight to tibia length was measured (A) 1 day (n = 8 per group), (B) 3 days (n = 4–5 per group), or (C) 6 days (n = 4–6 per group) following the administration of DOX or saline in male or female mice. Values are expressed as mean ± SEM. * *P* < 0.05, compared to saline-treated mice of the same sex; # *P* < 0.05, compared to male mice of the same treatment by Tukey’s post-hoc analysis.

**Fig 2:**
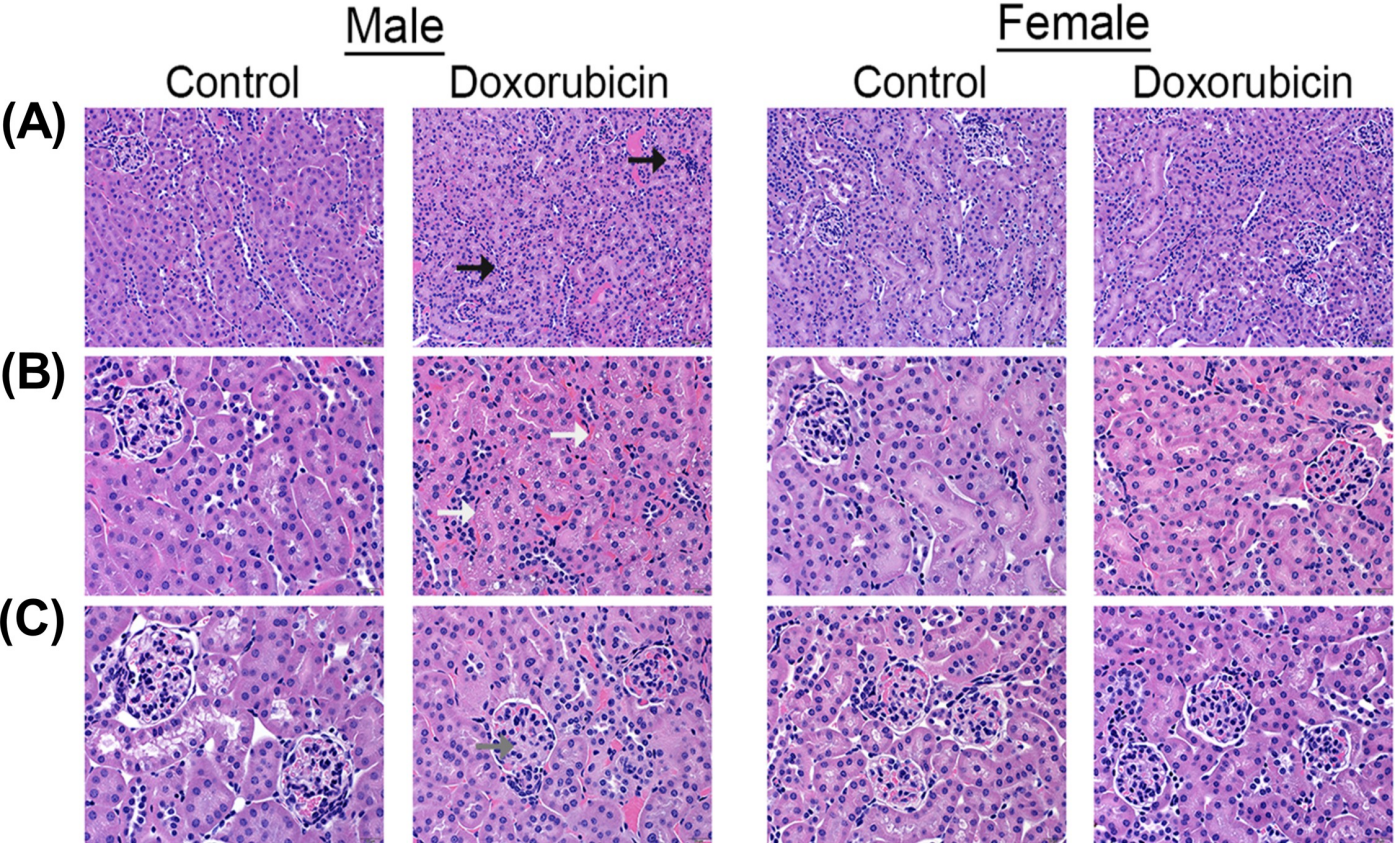
Effect of acute DOX administration on kidney histology. The kidneys harvested from adult male and female C57Bl/6 mice 6 days following administration of a single intraperitoneal injection of 20 mg/kg DOX or equivalent volume of sterile saline (control) were evaluated on hematoxylin and eosin stained sections. The DOX-treated male mice demonstrated mild inflammatory and degenerative renal pathology characterized by mild lymphocytic interstitial inflammation (black arrows), mild to moderate tubular degeneration (white arrow), and increased glomerular mesangial matrix (gray arrow). No such pathology was identified in the male control mice or either group of female mice.

**Fig 3:**
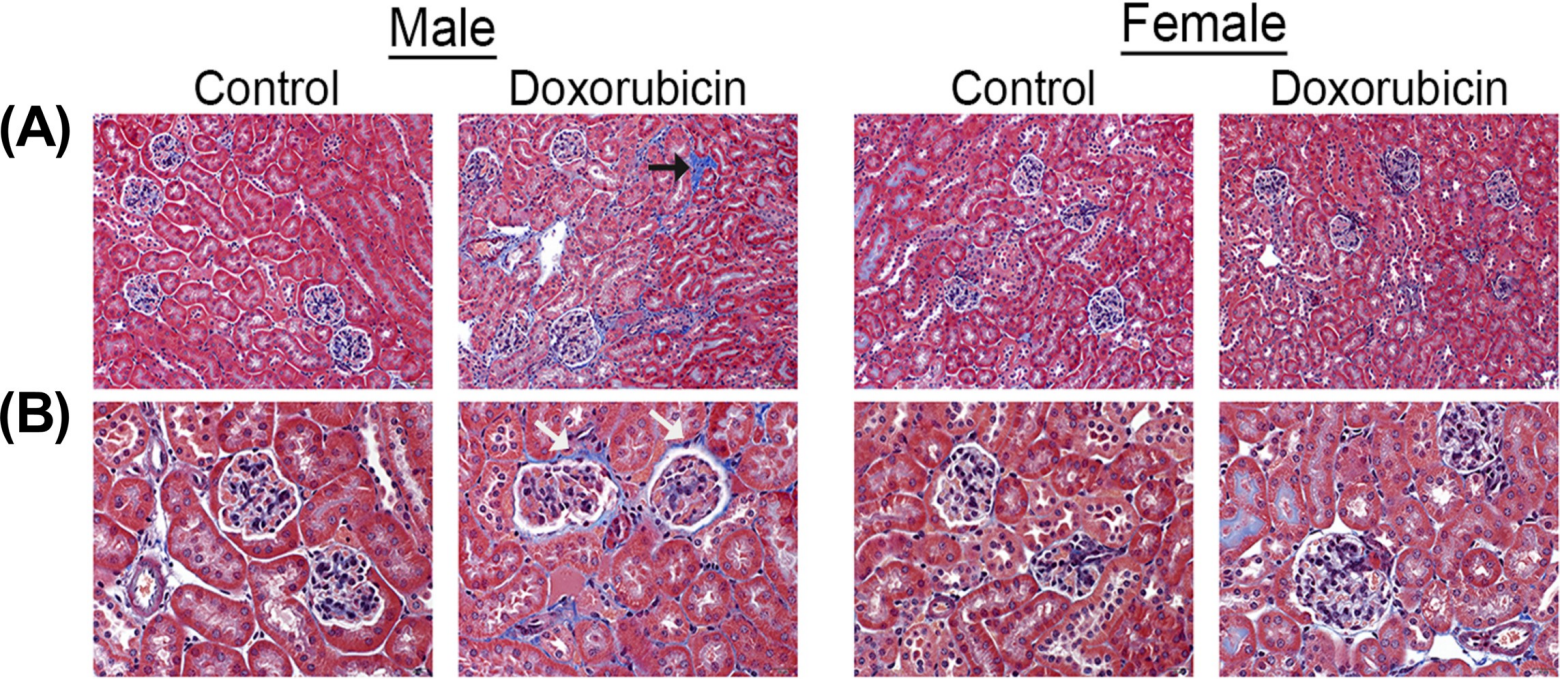
Effect of acute DOX administration on kidney fibrosis. The kidneys harvested from adult male and female C57Bl/6 mice 6 days following administration of a single intraperitoneal injection of 20 mg/kg DOX or equivalent volume of sterile saline (control) were evaluated for fibrosis using Masson’s trichrome stain. The DOX-treated male mice demonstrated increased deposition of collagen (blue) in both the interstitial space (black arrow) and surrounding glomeruli (white arrows). No such pathology was identified in the male control mice or either group of female mice.

**Fig 4:**
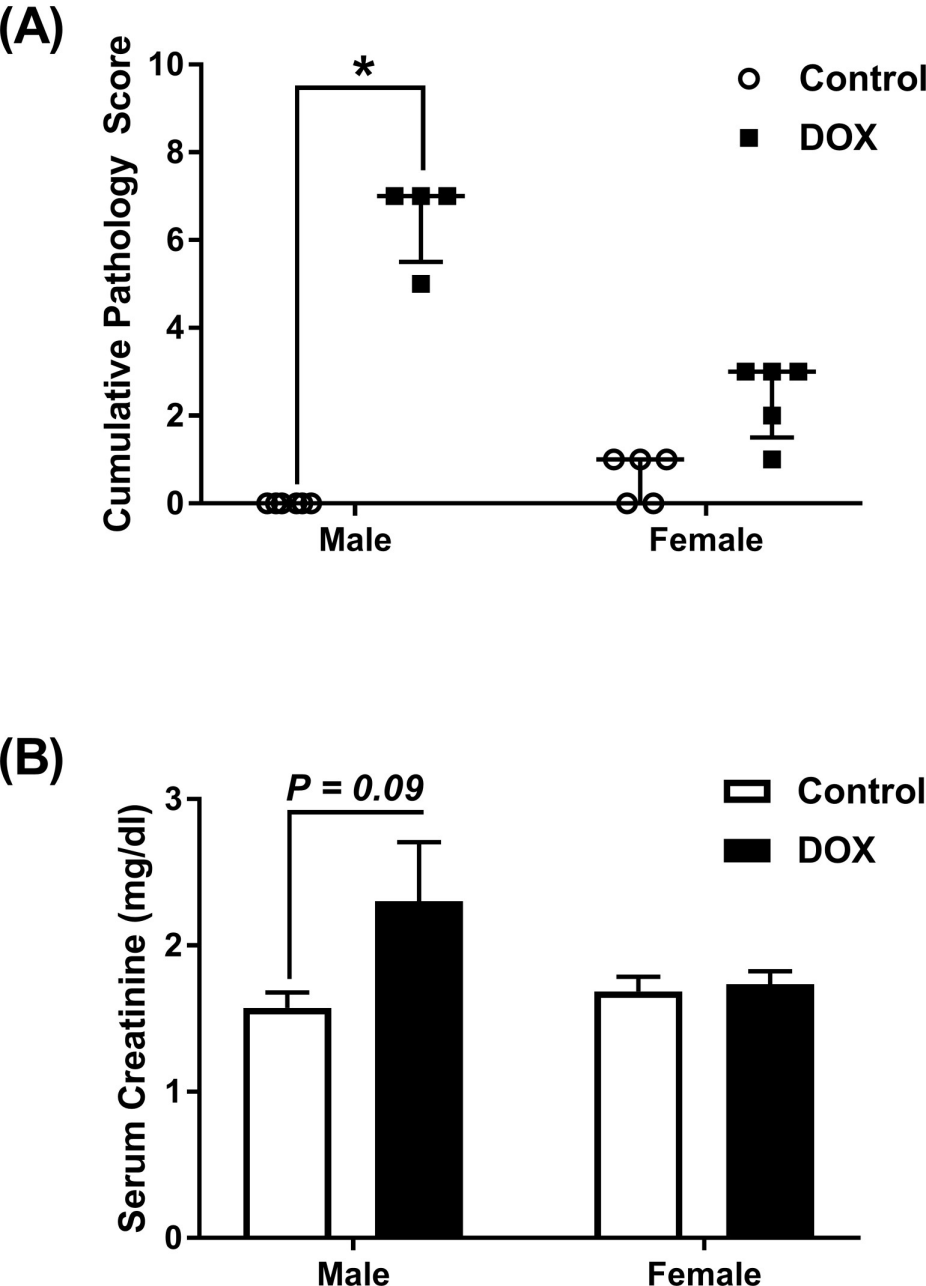
Effect of acute DOX administration on cumulative pathology score and kidney function. Male and female C57Bl/6 mice were administered a single intraperitoneal injection of 20 mg/kg DOX or equivalent volume of sterile saline (control) and followed up 6 days later. (A) Cumulative kidney pathology score. Individual scores and the median values are shown with interquartile range. * *P* < 0.05, compared to saline-treated mice of the same sex by Kruskal-Wallis non-parametric test. (B) Serum creatinine values (n = 3 per group). Expressed values are mean ± SEM.

Kidney weights of female control mice were significantly smaller than male control mice (Fig 1A–1C). Acute administration of 20 mg/kg DOX resulted in a significant 10%, 21%, and 23% reduction in paired kidney weight to tibia length ratio 1 day (Fig 1A), 3 days (Fig 1B), and 6 days (Fig 1C), respectively, after DOX injection in male, but not in female, mice. To further assess DOX-induced nephrotoxicity, sections from the kidneys collected 1, 3, and 6 days from control and DOX-treated mice were stained by H&E and trichrome stains. There was no evidence of significant pathological lesions in the kidneys collected 1 and 3 days after DOX administration from both male and female mice (S1 Fig). H&E staining of kidney tissues collected 6 days after DOX administration show that DOX causes higher interstitial cellularity (Fig 2A), more severe tubular degeneration/vacuolization (Fig 2B), and higher glomerular matrix deposition (Fig 2C) in male DOX-treated mice as compared to control male mice. Similarly, trichrome staining shows more severe renal fibrosis in kidneys of male DOX-treated mice as evidenced by higher interstitial (Fig 3A) and capsular (Fig 3B) collagen deposition. The cumulative pathology score was significantly higher in DOX-treated male, but not female, mice as compared to the respective control (Fig 4A). In order to determine the effect of DOX-induced nephrotoxicity on kidney function, creatinine levels were measured in the serum collected from mice sacrificed 1, 3, and 6 days after DOX administration. There was no difference in serum creatinine levels between control and DOX-treated mice at 1 and 3 days following DOX administration (data not shown). Conversely, a 50% increase in the level of serum creatinine was evident 6 days following DOX treatment in male mice, with a P value of 0.09, but not in female mice (Fig 4B).

### Effect of acute DOX administration on the apoptotic marker *BAX* gene expression

**Fig 5:**
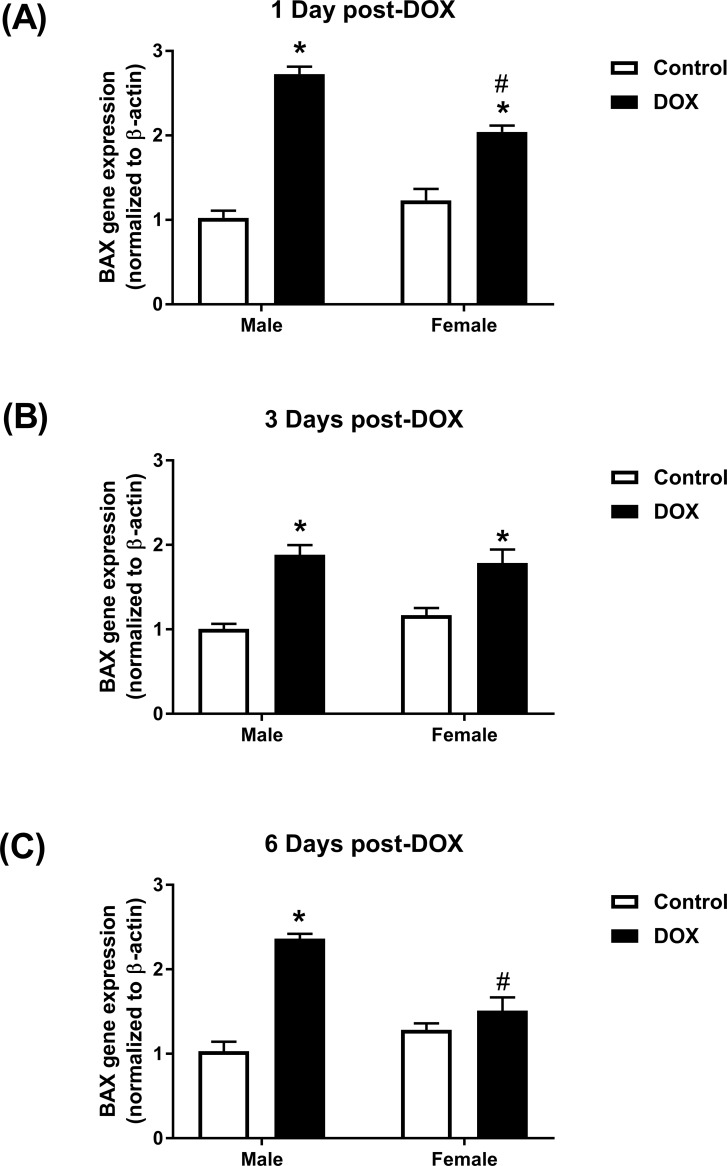
Effect of acute DOX administration on *BAX* gene expression. Kidneys were harvested from male or female C57Bl/6 mice (A) 1 day (n = 8 per group), (B) 3 days (n = 4–5 per group), or (C) 6 days (n = 4–6 per group) following the administration of 20 mg/kg DOX or equivalent volume of sterile saline and total RNA was prepared. *BAX* gene expression was determined by real-time PCR. Values were normalized to beta-actin and expressed relative to male control of the same time point. Values are expressed as mean ± SEM. * *P* < 0.05, compared to saline-treated mice of the same sex; # *P* < 0.05, compared to male mice of the same treatment by Tukey’s post-hoc analysis.

Similar basal levels of *BAX* gene expression were observed in male and female control kidneys (Fig 5A–5C). Acute DOX exposure caused a significant 2.7- and 2.0-fold induction of *BAX* gene expression in kidneys of male and female mice, respectively, 24 hours after DOX administration (Fig 5A). Similarly, a significant 1.9- and 1.8- fold induction of *BAX* gene expression was observed in male and female mice, respectively, 3 days after exposure to DOX (Fig 5B). Six days following DOX administration, *BAX* gene expression was similar to control values in kidneys of female mice, but was still induced in male mice by 2.4-fold (Fig 5C).

### Effect of acute DOX administration on the fibrotic marker transforming growth factor- β (TGF-β)

**Fig 6:**
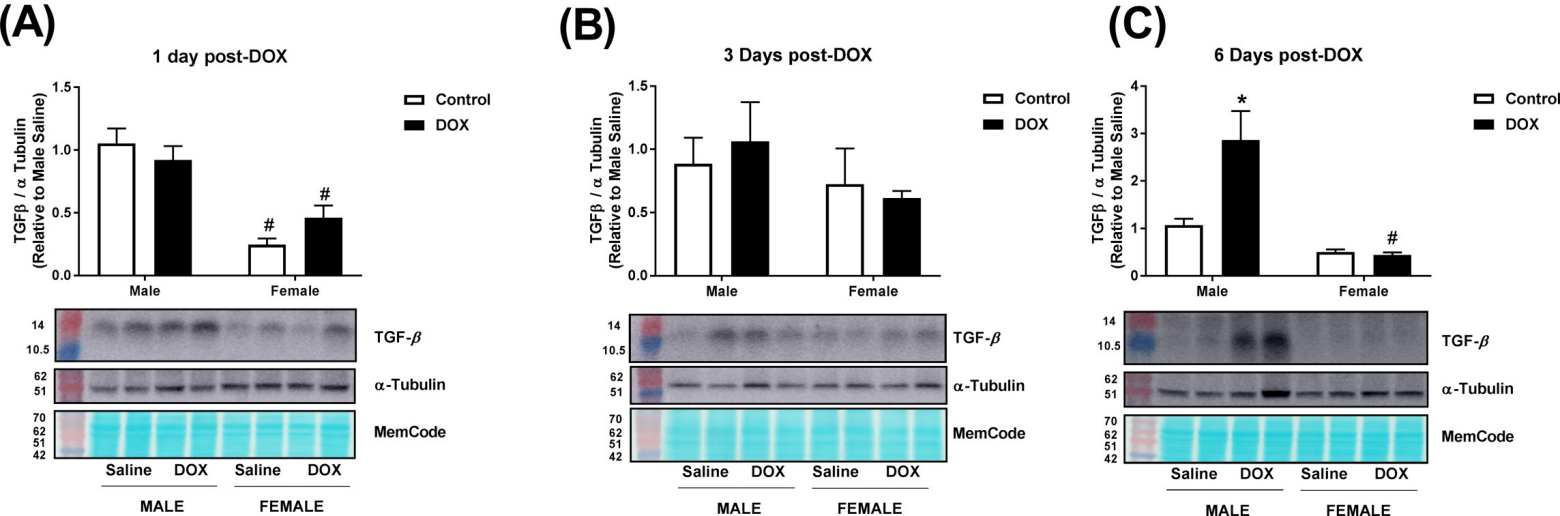
Effect of acute DOX exposure on TGF-β protein expression. Total protein was extracted from the kidneys of male or female C57Bl/6 mice (A) 1 day (n = 6 per group), (B) 3 days (n = 4–5 per group) or (C) 6 days (n = 4–6 per group) following the administration of 20 mg/kg DOX or equivalent volume of sterile saline. Quantification and representative immunoblots of TGF-β and alpha-tubulin are shown. MemCode total protein stain illustrates equal loading. Values were normalized to alpha-tubulin and are expressed relative to male control (n = 4–6 per group). Values are expressed as mean ± SEM. * *P* < 0.05, compared to saline-treated mice of the same sex; # *P* < 0.05, compared to male mice of the same treatment by Tukey’s post-hoc analysis.

Constitutive expression of TGF-β protein was 30–75% higher in male than in female control kidneys (Fig 6A–6C). No significant changes in TGF-β protein expression were observed 1 or 3 days following DOX administration (Fig 6A and 6B). However, 6 days following DOX administration, there was a significant 2.7-fold induction of TGF-β protein expression in kidneys of male, but not in female, mice (Fig 6C). Uncropped blots are shown in S2–S4 Figs.

### Acute DOX administration alters the expression of sEH in a time- and sex- dependent manner

**Fig 7:**
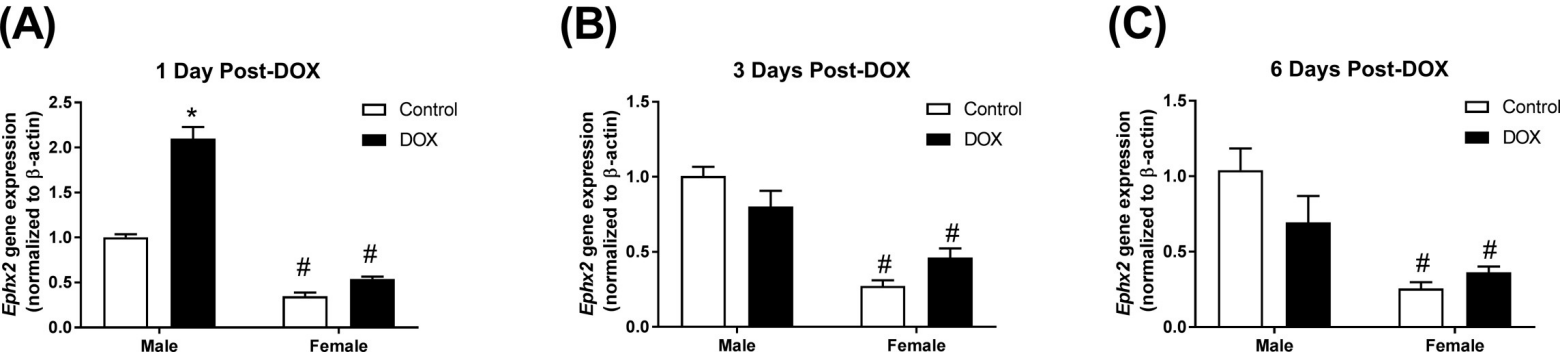
Effect of acute DOX administration on *Ephx2* gene expression. Kidneys were harvested from male or female C57Bl/6 mice (A) 1 day (n = 8 per group), (B) 3 days (n = 4–5 per group), or (C) 6 days (n = 4–6 per group) following the administration of 20 mg/kg DOX or equivalent volume of sterile saline and total RNA was prepared. *Ephx2* gene expression was determined by real-time PCR. Values were normalized to beta-actin and expressed relative to male control of the same time point. Values are expressed as mean ± SEM. * *P* < 0.05, compared to saline-treated mice of the same sex; # *P* < 0.05, compared to male mice of the same treatment by Tukey’s post-hoc analysis.

**Fig 8:**
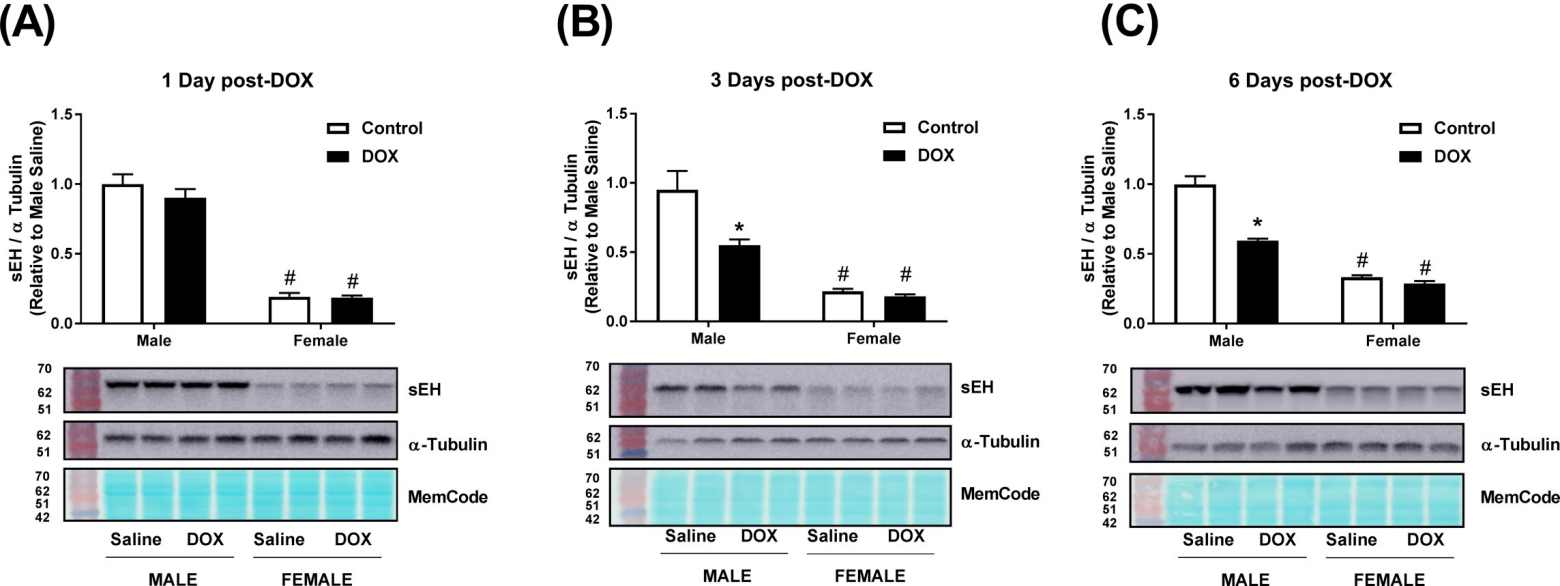
Changes in sEH expression following acute DOX administration. Total protein was extracted from the kidneys of male or female C57Bl/6 mice (A) 1 day (n = 6 per group), (B) 3 days (n = 4–5 per group) or (C) 6 days (n = 4–6 per group) following the administration of 20 mg/kg DOX or equivalent volume of sterile saline. Quantification and representative immunoblots of sEH and alpha-tubulin are shown. MemCode total protein stain illustrates equal loading. Values were normalized to alpha-tubulin and are expressed relative to male control (n = 4–6 per group). Values are expressed as mean ± SEM. * *P* < 0.05, compared to saline-treated mice of the same sex; # *P* < 0.05, compared to male mice of the same treatment by Tukey’s post-hoc analysis.

**Fig 9:**
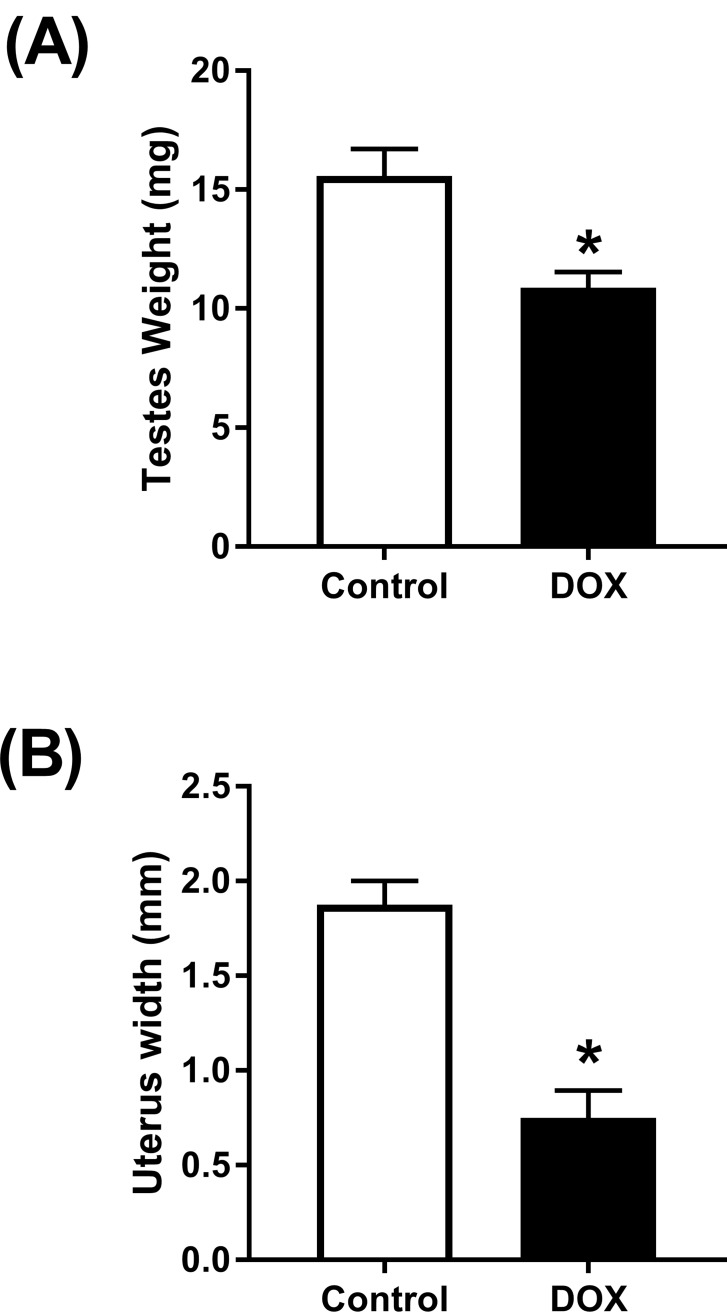
Effect of acute DOX administration on reproductive organs. (A) Testes weights or (B) uterus widths were measured 3 days (n = 4–5 per group) following the administration of a single injection of 20 mg/kg DOX or saline in male or female mice, respectively. Values are expressed as mean ± SEM. * *P* < 0.05, compared to saline-treated mice by unpaired t-test.

The constitutive expressions of *Ephx2*, the gene encoding sEH, and sEH protein were significantly lower in female than in male kidneys (Figs 7A–7C and 9A–9C). A significant 2.1-fold increase in the gene expression of *Ephx2* in kidneys of male, but not female, mice was observed 1 day following acute DOX administration (Fig 7A). Three and six days following acute DOX exposure, a trend toward lower *Ephx2* gene expression was observed in male, but not in female, mice (Fig 7B and 7C). No significant changes were observed in renal sEH protein expression in male or female mice 1 day following acute DOX administration (Fig 8A). Conversely, significant reductions in sEH protein expression were evident in kidneys of DOX-treated male mice compared to control male mice 3 days (45%) and 6 days (40%) after DOX administration, but not in female mice (Fig 8B and 8C). Uncropped blots are shown in S5–S7 Figs.

### Effect of acute DOX administration on reproductive organs

Compared to male control mice, acute DOX administration resulted in a 30% reduction in testes weight 3 days following treatment (Fig 9A). In female mice, acute DOX administration resulted in a 60% reduction in uterus width as compared to control female mice (Fig 9B).

### Effect of gonadectomy on renal sEH protein expression

**Fig 10:**
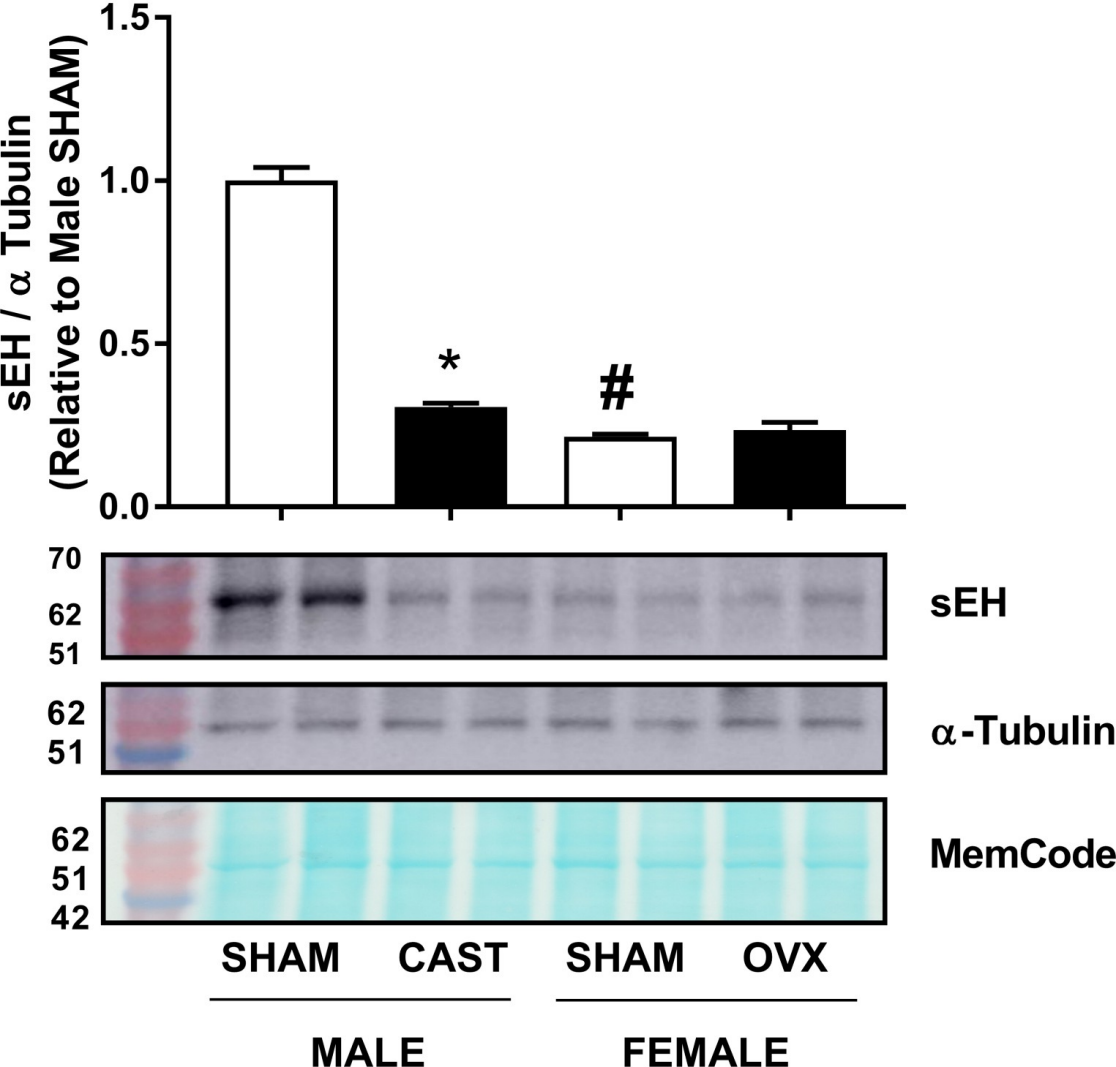
Effect of gonadectomy on renal sEH protein expression. sEH protein expression was measured in sham-operated, castrated male, or ovariectomized female mice. Quantification and representative immunoblots of sEH and alpha-tubulin. MemCode total protein stain illustrate equal loading. Values were normalized to alpha-tubulin and are expressed relative to male sham-operated mice (n = 4 per group). Values are expressed as mean ± SEM. * *P* < 0.05, compared to sham-operated mice of the same sex; # *P* < 0.05, compared to male mice of the same treatment by Tukey’s post-hoc analysis.

Male or female mice were castrasted or ovariectomized at 4 weeks of age and sEH protein expression was determined when mice reached 13 weeks of age. Sham-operated female mice expressed significantly lower amounts of sEH protein than sham-operated male mice (Fig 10). A significant 70% reduction in sEH protein expression was observed in male castrated mice as compared to sham-operated controls (Fig 10). In contrast, ovariectomy did not cause a significant change in renal sEH protein expression in female mice (Fig 10). Uncropped blots are shown in S8 Fig.

## Discussion

Sexual dimorphism of DOX-induced nephrotoxicity has been reported in a number of studies which demonstrated that female rats are protected [3, 6, 16, 17]. Nevertheless, sex-related differences in DOX-induced nephrotoxicity are far less documented in mice, despite the wide use of DOX-induced nephropathy as a murine model of proteinuric renal disease [18–20]. In a single study, Si *et al*. showed that female BALB/c mice were protected against the nephrotoxic effect of a single intra-venous dose of DOX (12 mg/kg) [21]. In agreement with these studies, we demonstrate that acute administration of a single intra-peritoneal dose of DOX (20 mg/kg) to C57Bl/6 mice caused significantly more severe glomerular and tubular lesions and renal fibrosis in the kidneys of male mice as compared to females.

In an attempt to elucidate the underlying mechanisms of this sexual dimorphism, we sought to determine the temporal associations between DOX-induced changes in molecular markers of nephrotoxicity and DOX-induced pathological and functional changes. To this end, we studied DOX-induced nephrotoxicity in male and female C57Bl/6 mice at three time points: 1, 3, and 6 days following DOX administration. With respect to DOX-induced molecular changes, we determined sex-related differences in DOX-induced upregulation of the apoptotic marker *BAX*, and the fibrotic marker TGF-β. DOX has been reported to induce *BAX* expression in kidneys of male rodents, and protection against DOX-induced nephrotoxicity was associated with inhibition of *BAX* expression [22–24]. In the current work, we demonstrate that DOX upregulated *BAX* gene expression in kidneys of male and female mice as early as 1 day after DOX administration. Six days after DOX administration, there was a significant upregulation of *BAX* gene expression in kidneys of male, but not in female, mice, suggesting a significant sex-related difference in DOX-induced apoptosis. In contrast to the early upregulation of the apoptotic marker *BAX*, there was no difference in the renal expression of the fibrotic marker TGF-β, in tissue histopathology, or in serum creatinine level between control and DOX-treated mice 1 and 3 days after DOX administration in both males and females, demonstrating that DOX-induced apoptosis precedes pathological and functional changes. Conversely, DOX-induced pathological lesions and increased serum creatinine were observed in male, but not female, mice sacrificed 6 days after DOX administration. In agreement with the higher fibrosis in kidneys of DOX-treated male mice, the protein expression of TGF-β was significantly higher in kidneys of DOX-treated male, but not female, mice 6 days following DOX administration. The increase in TGF-β expression has been previously demonstrated to be critical in the pathogenesis of DOX-induced renal fibrosis in male experimental animals [5, 25]. To the best of our knowledge, however, this is the first report of a sex-related difference in DOX-induced TGF-β expression in the kidney. Intriguingly, we also observed a lower constitutive level of TGF-β in kidneys of female mice than in males, which may explain, at least in part, the marked sexual dimorphism in DOX-induced renal fibrosis.

A number of studies have reported the effect of DOX and other nephrotoxic chemotherapies on sEH expression in kidney tissues with discrepant findings. In kidneys of adult male Sprague Dawley rats, we previously demonstrated that a single intra-peritoneal injection of DOX (15 mg/kg) inhibited sEH protein expression 1 day following DOX administration without a significant change in *Ephx2* gene expression [26], suggesting that the immediate effect of acute DOX administration is to inhibit renal sEH expression. Similarly, both acute arsenic- and cisplatin-induced nephrotoxicity have been associated with inhibition of sEH protein expression 1 and 3 days after DOX administration, respectively [27, 28]. Contrariwise, a single tail-vein injection of DOX (10 mg/kg) has been reported to upregulate sEH protein expression in the kidney of adult male BALBc mice 2 and 6 weeks following DOX administration [9]. Similarly, a single tail-vein injection of DOX (7 mg/kg) upregulated sEH protein expression in the kidney of adult male Wistar rats 6 weeks after DOX administration [8], suggesting that the delayed effect of acute DOX administration is to induce sEH expression. Nevertheless, all the aforementioned studies had two limitations: first, they were conducted in male rodents only; second, they did not study the temporal association between DOX-induced changes in sEH expression and kidney pathology. In order to address these issues, we measured gene and protein expression of sEH in male and female mice 1, 3, and 6 days after DOX administration. In the current study, we demonstrate that the gene expression of *Ephx2*, the gene encoding sEH, was induced in the kidney tissue of male DOX-treated mice, but not in female mice, 1 day after DOX administration. However, there was no difference in renal sEH protein expression between control and DOX-treated mice in male or female mice. Discrepancy between gene and protein expression is not uncommon, and can be explained by post-transcriptional and post-translational modifications [26, 29]. Since the induction of gene expression precedes the translation of the mRNA to protein, there was a possibility that the induction of *Ephx2* gene expression 1 day following DOX administration would result in an increase in sEH protein expression at a later time point. To test this hypothesis, we measured sEH protein expression in kidneys of two cohorts of male and female mice 3 and 6 days after DOX administration. Counterintuitively, sEH protein expression was significantly lower in the kidney of male DOX-treated mice than in control mice, while there was no significant difference in females 3 and 6 days after DOX administration. Although we did not measure renal sEH activity in the current study, sEH protein expression has been shown to be well correlated with its activity [11, 27, 30]. These findings clearly demonstrate that DOX-induced nephrotoxicity in male mice was not mediated by induction of the sEH enzyme. Considering the known detrimental effects of sEH on the kidney [30], inhibition of sEH may be involved in a negative feedback mechanism to protect the kidney from further DOX-induced injury, similar to what has been reported in the context of acute cisplatin-induced nephrotoxicity [27].

Nevertheless, the mechanism by which DOX inhibits sEH is still obscure. The direct effects of DOX in the kidney, e.g. DOX-induced oxidative stress and proteinuria, have previously been shown to induce sEH, not to inhibit it [9, 31]. Therefore, DOX may have inhibited sEH in an indirect mechanism. Indeed, acute DOX administration causes multi-organ toxicity with potential interplay between different organs to alter sEH expression. Importantly, DOX administration has been shown to cause significant testicular toxicity and marked decrease in serum testosterone in experimental animals [32–36]. In agreement with these studies, we found out that acute DOX administration caused a significant reduction of the testes weights in male mice 3 days after DOX administration, indicating DOX-induced testicular toxicity. We also demonstrate that castration of male mice markedly inhibited renal sEH, in agreement with previous reports [11]. Taken together, DOX-induced testicular toxicity and the resulting low testosterone level may have indirectly inhibited renal sEH. Although intriguing, this hypothesis needs to be confirmed in future studies by administering testosterone to DOX-treated male mice. In female mice, we also demonstrate that acute DOX administration caused a significant reduction in uterus widths, indicating DOX-induced ovarian suppression as previously reported [37, 38]. Interestingly, ovariectomy of female mice did not alter the expression of renal sEH protein expression. The interplay between DOX-induced testicular toxicity and inhibition of renal sEH can also explain the inhibition of renal sEH by cisplatin and arsenic, because they have also been shown to cause testicular toxicity [39–41].

Importantly, the constitutive *Ephx2* gene and sEH protein expressions were consistently lower in the kidney of female mice than in males, in agreement with earlier studies [11, 12]. Indeed, the biological importance of this observation had not been recognized until recently, when a sex-related difference in sEH expression was proposed to explain sex differences in ischemic brain injury [42], pulmonary hypertension [43], flow-mediated dilation of microvessels [44], and cardiac function [45]. Although the current work demonstrates that induction of sEH is not necessary for the initiation of acute DOX-induced nephrotoxicity, low constitutive expression of renal sEH and the expected increase in EETs may have conferred protection against DOX-induced apoptotic and fibrotic effects in female mice. This notion is supported by several lines of evidence: first, pharmacologic and genetic inhibition of sEH has been shown to protect against DOX-induced nephrotoxicity [8, 9]; second, inhibition of sEH has been shown to protect against other models of nephropathy [30, 46]; third, EETs have both anti-apoptotic [47] and anti-fibrotic [48] properties in the kidney. Therefore, low constitutive expression of sEH in the female kidney may, at least in part, contribute to the sexual dimorphism of DOX-induced nephrotoxicity. Further research is required to comprehensively elucidate other sex-related differences that may have contributed to the observed sexual dimorphism including a detailed pharmacokinetic and tissue distribution analysis of DOX in male and female mice to determine whether higher exposure of the renal tissue to DOX in male mice may also play a role in sexual dimorphism in DOX-induced nephrotoxicity.

In conclusion, the current work demonstrates significant sex-related differences in molecular determinants and phenotypic manifestations of DOX-induced nephrotoxicity in C57Bl/6 mice. Lower constitutive expressions of TGF-β and sEH in the kidney of female mice may have conferred protection against DOX-induced nephrotoxicity, which may explain, at least in part, the observed sexual dimorphism. Studying the temporal association between sEH expression and DOX-induced pathological changes in the kidney revealed that induction of sEH is not required for the initiation of DOX-induced nephrotoxicity. Importantly, the current study suggests a possible interplay between DOX-induced gonadal toxicity and inhibition of renal sEH in DOX-treated male mice.

## Supporting information

S1 Fig Effect of acute DOX administration on kidney histology 1 and 3 days following administration. The kidneys harvested from adult male and female C57Bl/6 mice 1 and 3 days following administration of a single intraperitoneal injection of 20 mg/kg DOX or equivalent volume of sterile saline (control) were evaluated on (A) hematoxylin and eosin and (B) trichrome-stained sections.
(PDF)

S2 Fig Uncropped blots used for quantification in Fig 6A. Cropped area shown in Fig 6A is outlined with a black rectangle.
(PDF)

S3 Fig Uncropped blots used for quantification in Fig 6B. Cropped area shown in Fig 6B is outlined with a black rectangle.
(PDF)

S4 Fig Uncropped blots used for quantification in Fig 6C. Cropped area shown in Fig 6C is outlined with a black rectangle.
(PDF)

S5 Fig Uncropped blots used for quantification in Fig 8A. Cropped area shown in Fig 8A is outlined with a black rectangle.
(PDF)

S6 Fig Uncropped blots used for quantification in Fig 8B. Cropped area shown in Fig 8B is outlined with a black rectangle.
(PDF)

S7 Fig Uncropped blots used for quantification in Fig 8C. Cropped area shown in Fig 8C is outlined with a black rectangle.
(PDF)

S8 Fig Uncropped blots used for quantification in Fig 10. Cropped area shown in Fig 10 is outlined with a black rectangle.
(PDF)
