# Supplementary figures and images for: Sexual dimorphism of acute doxorubicin-induced nephrotoxicity in C57Bl/6 mice
Source: PLoS One. 2019 Feb 20;14(2):e0212486. doi: 10.1371/journal.pone.0212486 (PMC6382134; doi:10.1371/journal.pone.0212486)

S1 Fig

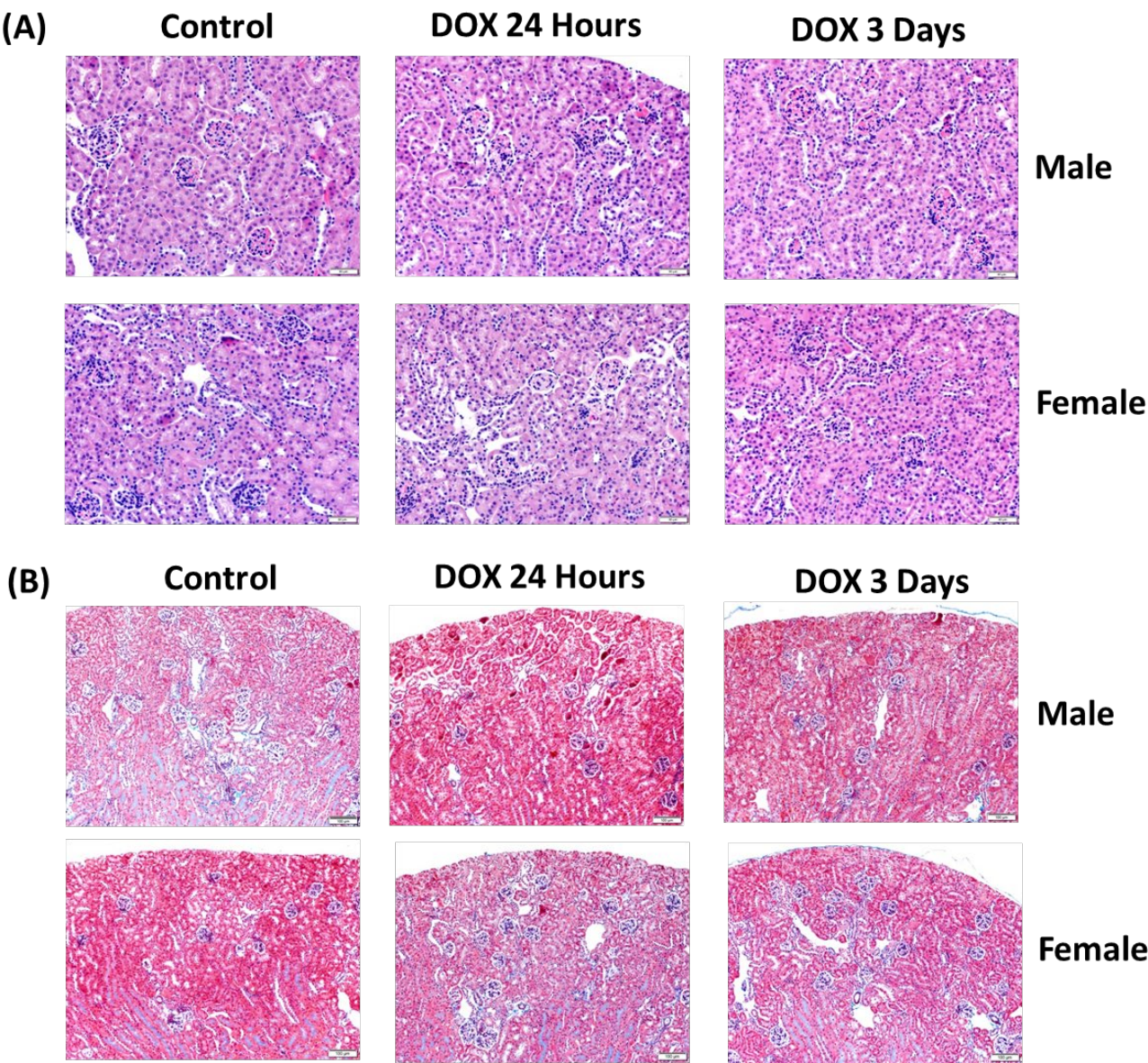

Supplement: S1 Fig — The kidneys harvested from adult male and female C57Bl/6 mice 1 and 3 days following administration of a single intraperitoneal injection of 20 mg/kg DOX or equivalent volume of sterile saline (control) were evaluated on (A) hematoxylin and eosin and (B) trichrome-stained sections. (PDF) [file pone.0212486.s001.pdf]

S2 Fig

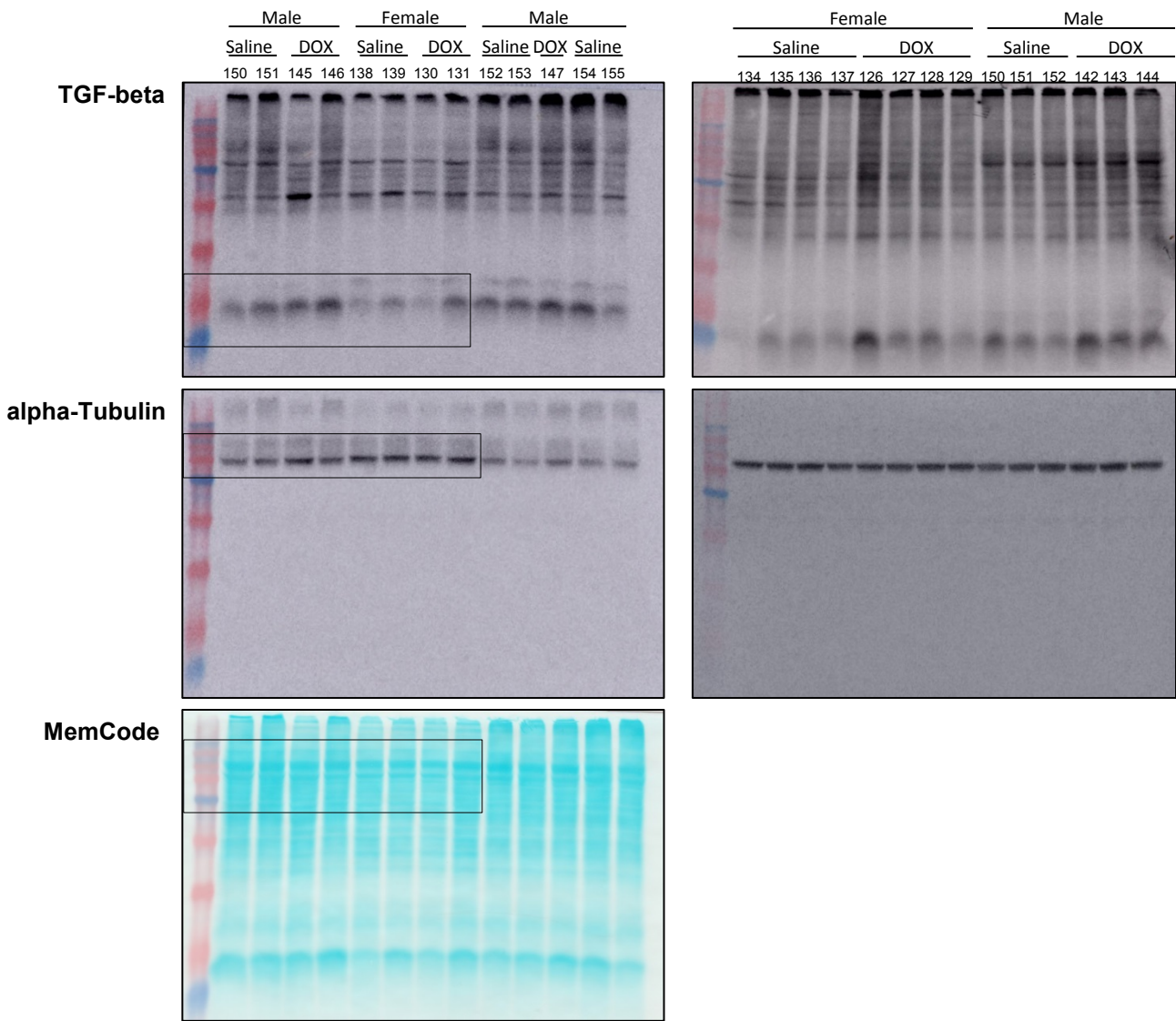

Supplement: S2 Fig — Cropped area shown in Fig 6A is outlined with a black rectangle. (PDF) [file pone.0212486.s002.pdf]

S3 Fig

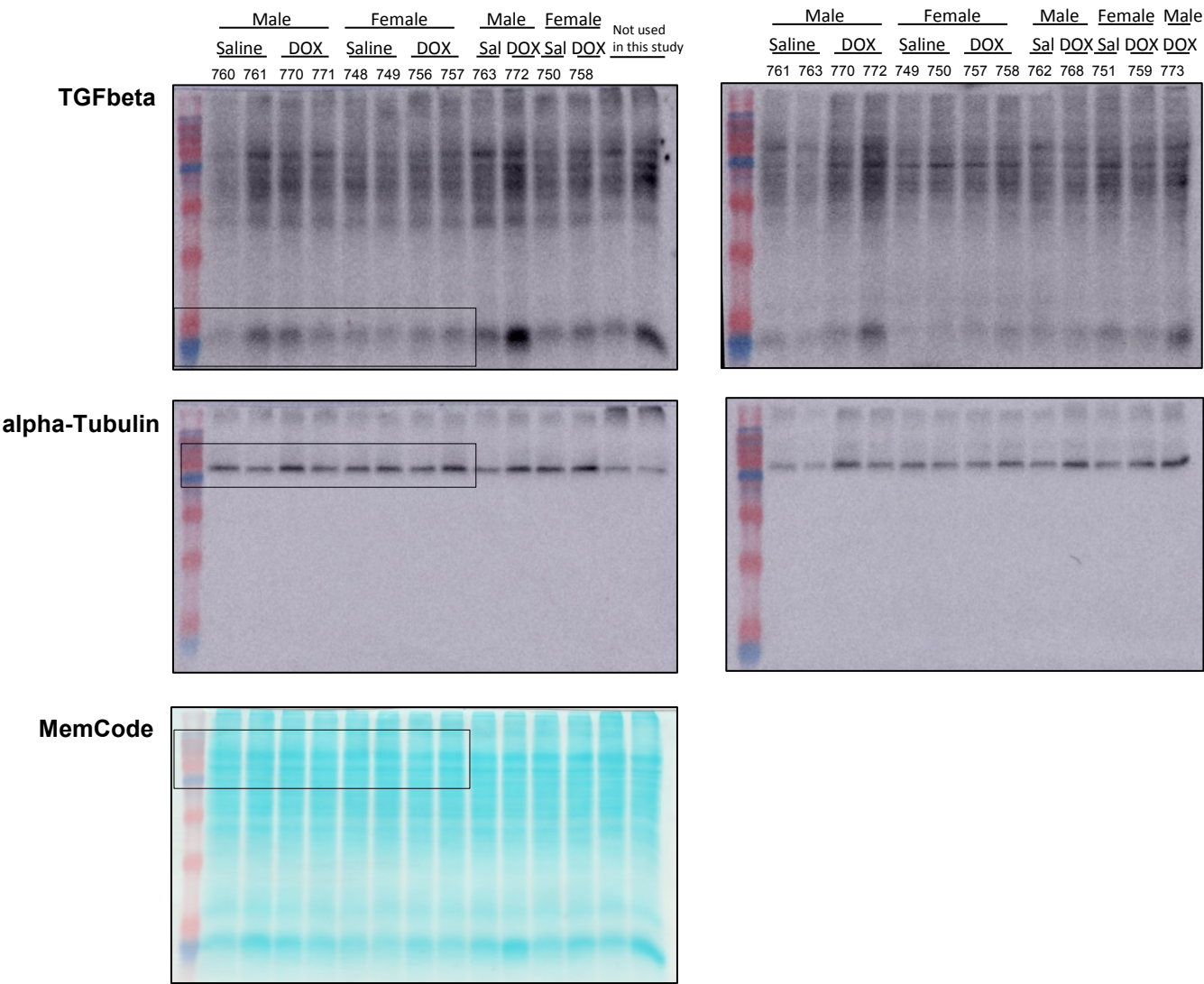

Supplement: S3 Fig — Cropped area shown in Fig 6B is outlined with a black rectangle. (PDF) [file pone.0212486.s003.pdf]

S4 Fig

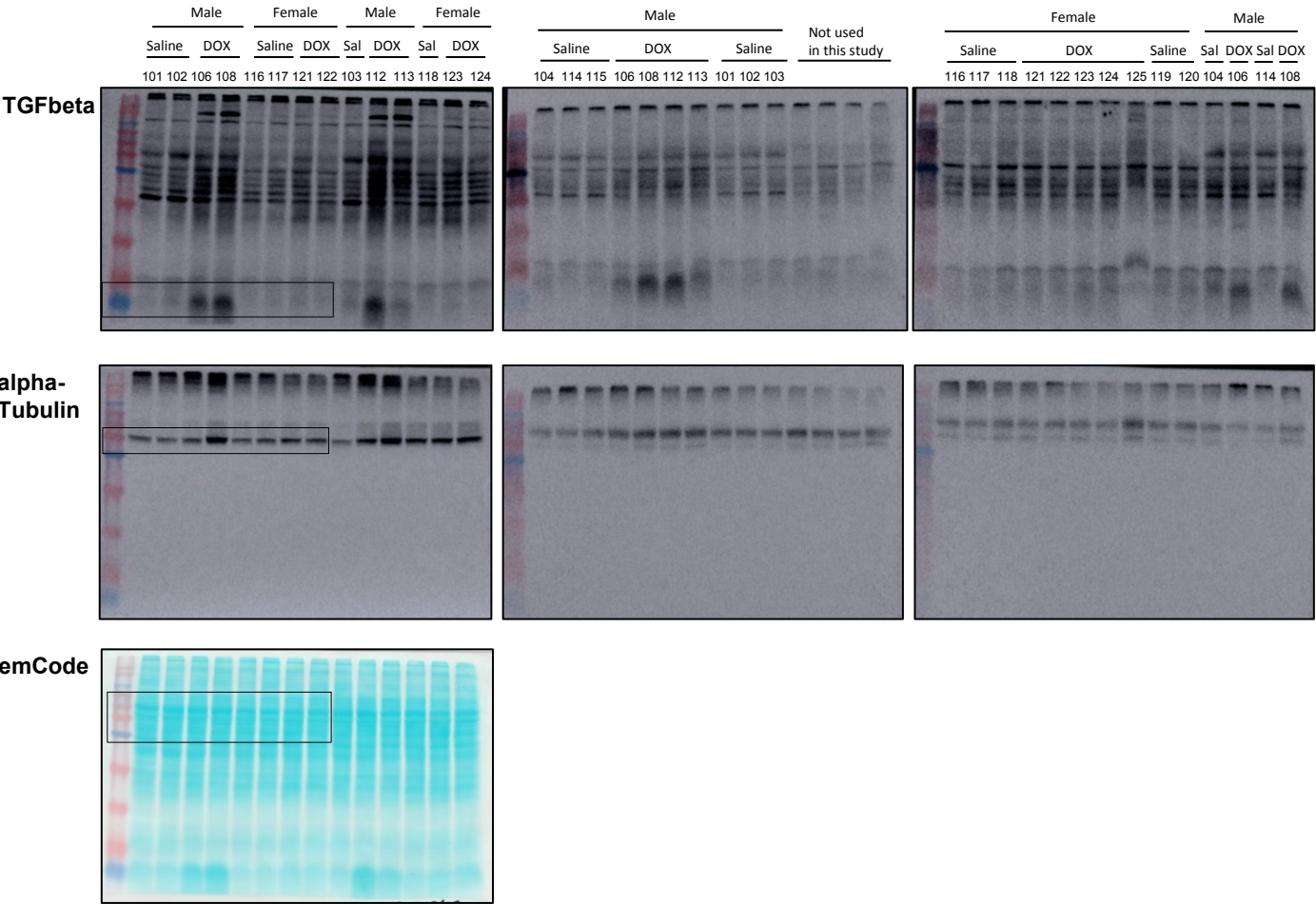

Supplement: S4 Fig — Cropped area shown in Fig 6C is outlined with a black rectangle. (PDF) [file pone.0212486.s004.pdf]

S5 Fig

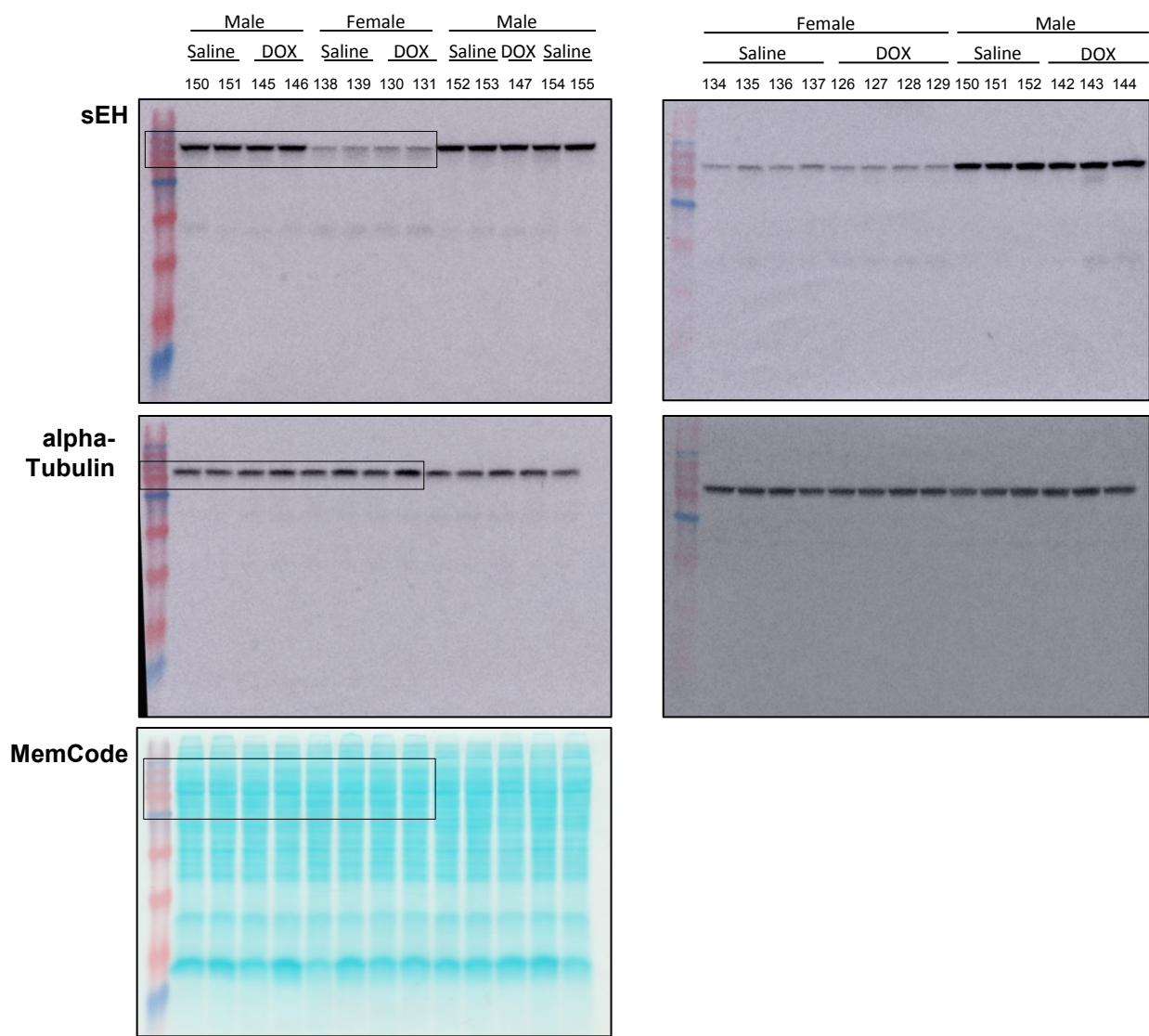

Supplement: S5 Fig — Cropped area shown in Fig 8A is outlined with a black rectangle. (PDF) [file pone.0212486.s005.pdf]

S6 Fig

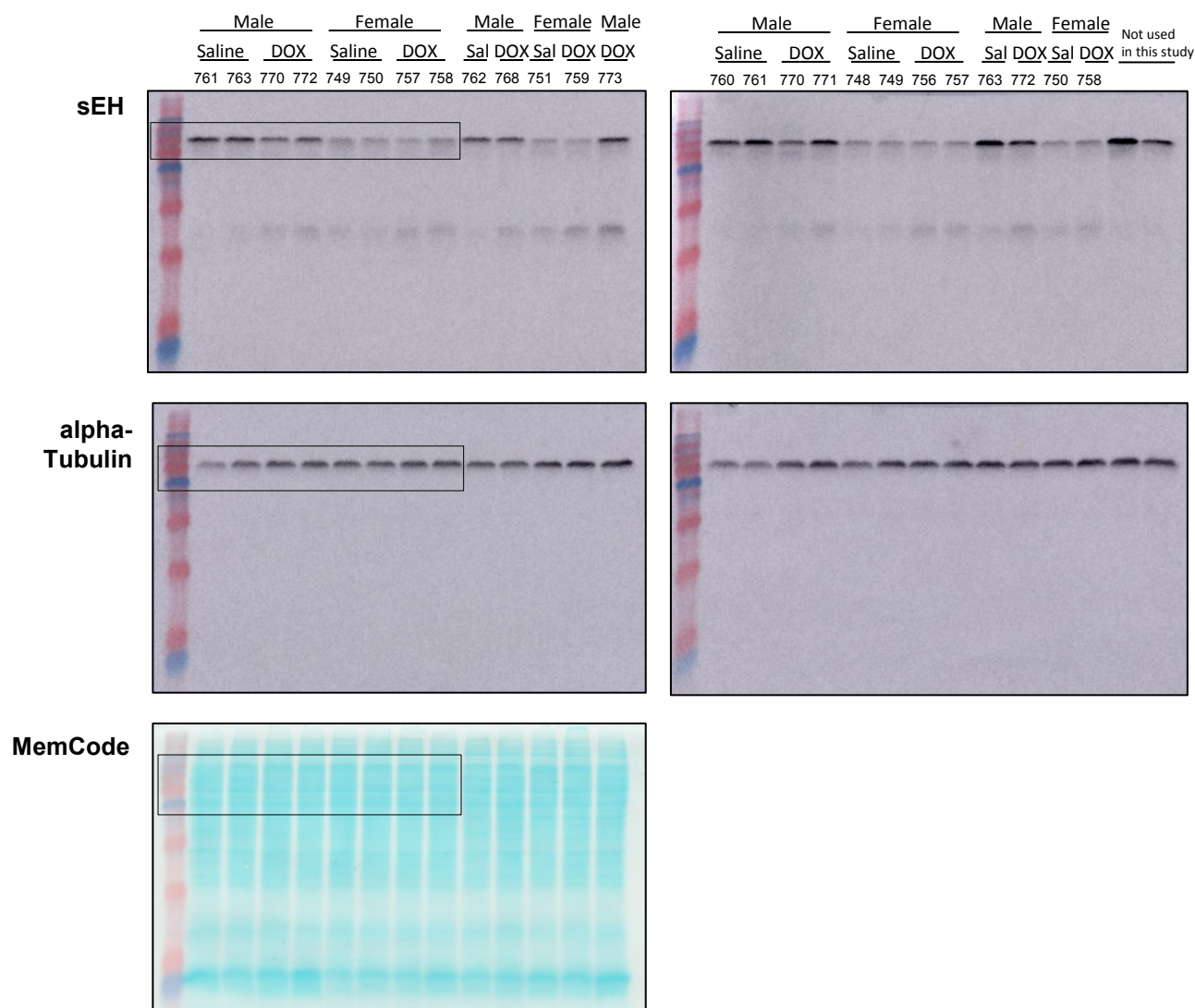

Supplement: S6 Fig — Cropped area shown in Fig 8B is outlined with a black rectangle. (PDF) [file pone.0212486.s006.pdf]

S7 Fig

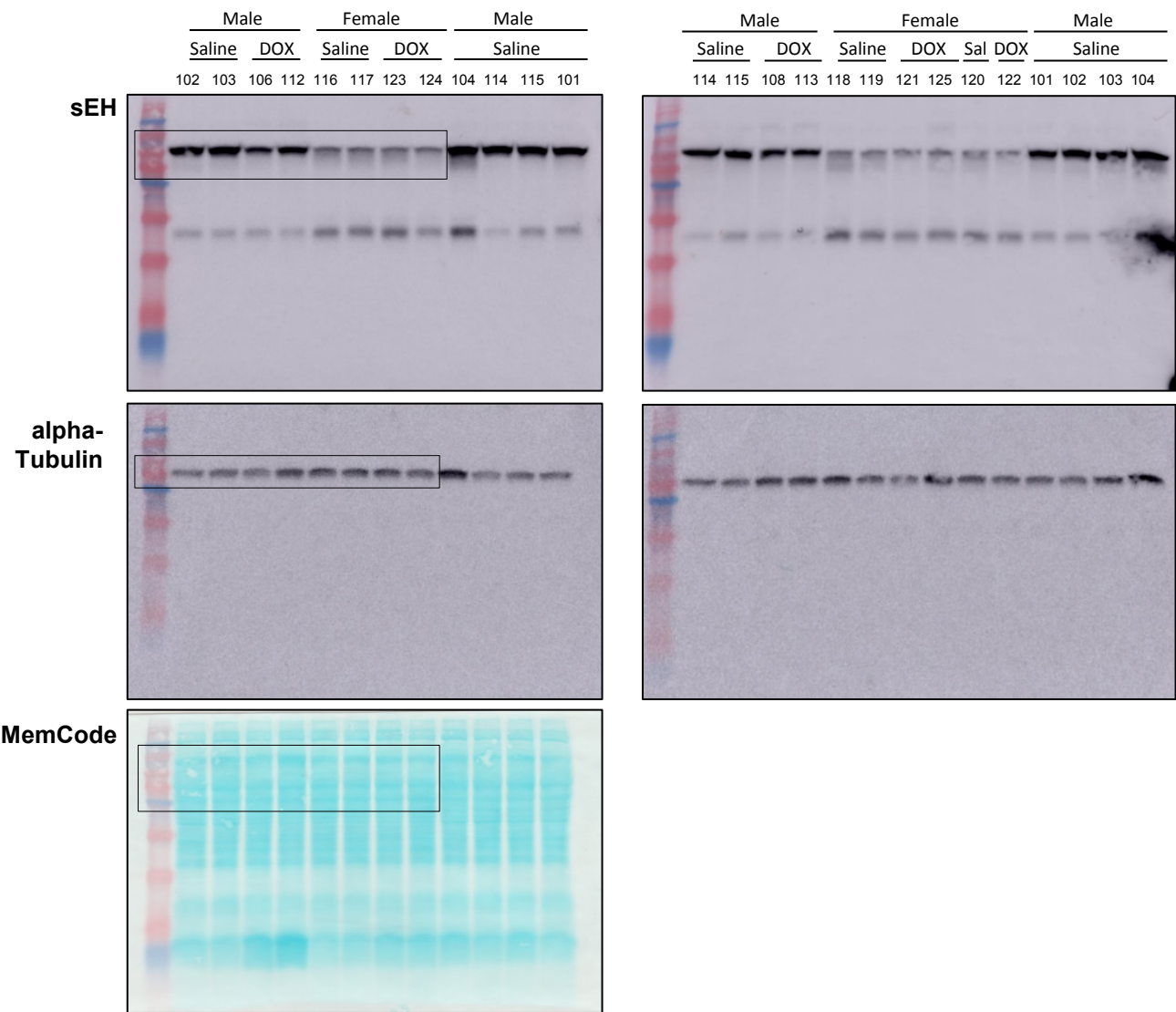

Supplement: S7 Fig — Cropped area shown in Fig 8C is outlined with a black rectangle. (PDF) [file pone.0212486.s007.pdf]

S8 Fig

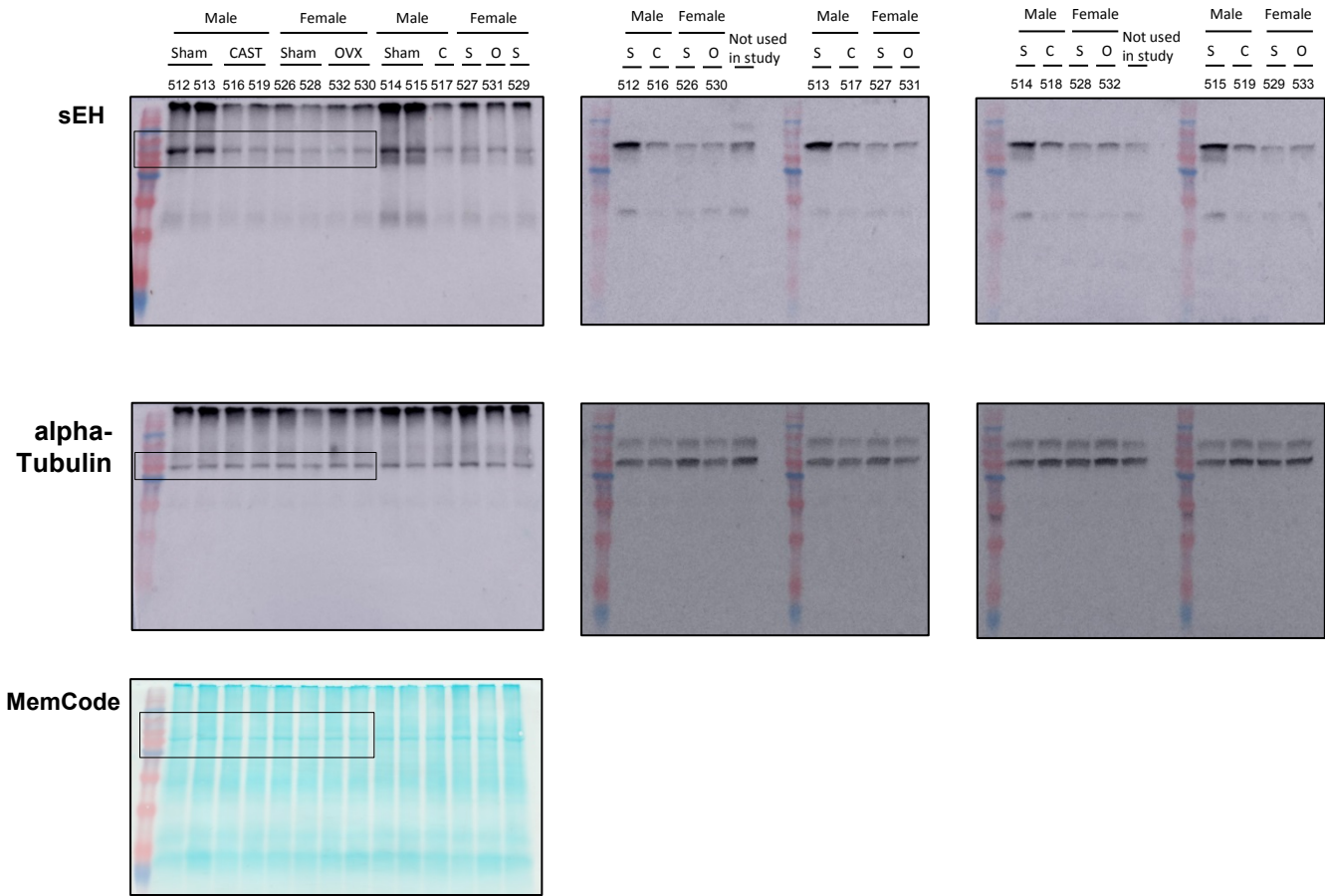

Supplement: S8 Fig — Cropped area shown in Fig 10 is outlined with a black rectangle. (PDF) [file pone.0212486.s008.pdf]
